# Mechanosensitive molecular interactions in atherogenic regions of the arteries: development of atherosclerosis

**DOI:** 10.18699/VJ21.062

**Published:** 2021-09

**Authors:** E.L. Mishchenko, A.M. Mishchenko, V.A. Ivanisenko

**Affiliations:** Institute of Cytology and Genetics of the Siberian Branch of the Russian Academy of Sciences, Novosibirsk, Russia; Novosibirsk State University, Novosibirsk, Russia; Institute of Cytology and Genetics of the Siberian Branch of the Russian Academy of Sciences, Novosibirsk, Russia

**Keywords:** atherogenesis, shear stress, transcription factor NF-κB;, RelA expression, mechanosensitive receptors, cell adhesion molecules, signaling pathways, mechanotransduction, атерогенез, напряжение сдвига, транскрипционный фактор NF-κB, экспрессия RelA, механочувствительные рецепторы, молекулы клеточной адгезии, сигнальные пути, механотрансдукция

## Abstract

A terrible disease of the cardiovascular system, atherosclerosis, develops in the areas of bends and branches of arteries, where the direction and modulus of the blood flow velocity vector change, and consequently so does the mechanical effect on endothelial cells in contact with the blood flow. The review focuses on topical research studies on the development of atherosclerosis – mechanobiochemical events that transform the proatherogenic mechanical stimulus of blood flow – low and low/oscillatory arterial wall shear stress in the chains of biochemical reactions in endothelial cells, leading to the expression of specific proteins that cause the progression of the pathological process. The stages of atherogenesis, systemic risk factors for atherogenesis and its important hemodynamic factor, low and low/oscillatory wall shear stress exerted by blood flow on the endothelial cells lining the arterial walls, have been described. The interactions of cell adhesion molecules responsible for the development of atherosclerosis under low and low/oscillating shear stress conditions have been demonstrated. The activation of the regulator of the expression of cell adhesion molecules, the transcription factor NF-κB, and the factors regulating its activation under these conditions have been described. Mechanosensitive signaling pathways leading to the expression of NF-κB in endothelial cells have been described. Studies of the mechanobiochemical signaling pathways and interactions involved in the progression of atherosclerosis provide valuable information for the development of approaches that delay or block the development of this disease. Key words: atherogenesis; shear stress; transcription factor NF-κB; RelA expression; mechanosensitive receptors; cell adhesion molecules; signaling pathways; mechanotransduction.

## Abstract (ru)

Атеросклероз, грозное заболевание сердечно-сосудистой системы, развивается в местах изгибов и разветвлений артерий, где меняются направление и модуль вектора скорости тока крови, а следовательно, механическое воздействие на контактирующие с током крови эндотелиальные клетки. Обзор посвящен актуальным исследованиям развития атеросклероза: механобиохимическим событиям, преобразующим проатерогенный механический стимул тока крови – низкое и низкое/осциллирующее напряжение сдвига, оказываемое на стенки артерий, – в цепи биохимических реакций в эндотелиальных клетках, приводящих к экспрессии специфичных белков, вызывающих прогрессирование патологического процесса. Описаны стадии, системные факторы риска, а также важный гемодинамический фактор атерогенеза: низкое и низкое/осциллирующее напряжение сдвига, оказываемое током крови на эндотелиальные клетки, выстилающие стенки артерий. Показаны взаимодействия молекул клеточной адгезии, ответственные за развитие атеросклероза в условиях низкого и низкого/осциллирующего напряжения сдвига. Описаны активация регулятора экспрессии молекул клеточной адгезии – транскрипционного фактора NF-κB – и факторы, контролирующие его активацию в этих условиях. Описаны механочувствительные сигнальные пути, приводящие к экспрессии NF-κB в эндотелиальных клетках. Исследования механобиохимических сигнальных путей и взаимодействий, вовлеченных в прогрессирование атеросклероза, необходимы для разработки подходов, задерживающих или блокирующих развитие заболевания.

## Risk factors and stages of atherogenesis.Shear stress is an important haemodynamic factor in atherogenesis

Nowadays, cardiovascular disease is a major public health issue. Moreover, atherosclerosis is one of the most common pathologies of the cardiovascular system. The systemic risk factors for the development of atherosclerosis include age, hypertension, diabetes mellitus, smoking, low physical activity, fatty diet, renal failure, increased level of fibrinogen, lowdensity lipoproteins, cholesterol and blood plasma C-reactive protein (Virani et al., 2020). The level of low-density lipoproteins (LDLs) is classified into a separate group of factors that account for the atherogenicity of the subfraction profile of apo-B-containing lipoproteins (Chang et al., 2017; Ozerova et al., 2018). The penetration of blood plasma LDLs through the endothelium in athero-susceptible areas of the arteries and their retention and accumulation in the extracellular matrix (ECM) of the subendothelial space initiates atherogenesis. LDLs are retained in the intima (mainly due to interaction with proteoglycans), undergo oxidation (formation of oxLDLs) and cause an inflammatory response – the infiltration of circulating blood monocytes into the intima. In the intima, monocytes differentiate into macrophages, uptake oxLDLs and become foam cells (Libby et al., 2019).

The development of atherosclerosis occurs in the following stages: (i) adaptive intimal thickening, (ii) formation of fatty streaks, (iii) pathological intimal thickening (PIT), (iv) early fibroatheroma and (v) late fibroatheroma. In stage (i), smooth muscle cells (SMCs) of the media migrate to the intima and secrete proteoglycans. The formation of fatty streaks in stage (ii) is accompanied by the accumulation of foamy cells (macrophages loaded with lipids) in the intima. Lipid-loaded SMCs are less represented. The PIT process (iii) occurs with and without the infiltration of macrophages. In both cases, SMCs and extracellular lipid pools are present in the intima. The accumulation of SMCs occurs towards the lumen of the artery, and lipid pools accumulate close to the media. The formation of a fibrous cap that covers the necrotic core occurs in the later stages of development of the atherosclerotic lesions, including early and late fibroatheroma (iv, v). The cap includes SMCs, infiltrated macrophages, T-lymphocytes, as well as collagens and proteoglycans of the extracellular matrix. Programmed cell death, via apoptosis and necroptosis, plays an essential role in early fibroatheroma (iv) with the formation of foci of necrosis and cholesterol crystals. In late fibroatheroma (v), an extensive necrotic core that consists of cellular debris and a large number of crystals of free cholesterol and its esters is formed (Otsuka et al., 2015).

The molecular and genetic processes of atherogenesis remain unclear. Numerous haemodynamic studies have shown that, based on systemic risk factors, atherosclerosis develops mainly in the bends and branching of the arteries, where there is a change in the nature of the blood flow (Cecchi et al., 2011; Morbiducci et al., 2016; Zou et al., 2016). The haemodynamic characteristics of the effect of blood flow on the vessel walls are wall shear stress (WSS), hydrostatic pressure and cyclic deformation. WSS is the friction force that occurs when flowing blood comes into contact with the inner wall of the artery. WSS on the arterial wall is described by (Ku et al., 1985):

**Form. 1.**
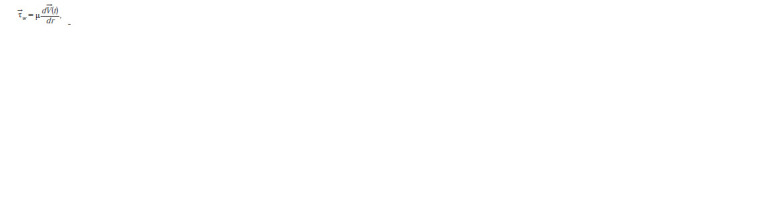
Form. 1.

where μ is the blood viscosity index, →V(t) is the blood flow rate parallel to the vessel wall at time t and r is the radial coor-dinate.

Studies conducted on animal models and observing patients revealed a regular maximum thickening of the intima and the formation of atherosclerotic plaques in areas with low WSS (< 10 dyn/cm2 in humans) and low/oscillatory WSS (with a deviation of the instantaneous WSS vector from its average direction). Such damage was minimal in areas of high WSS (>25 dyn/cm2 in humans). High values of WSS were realized in the rectilinear sections of the arteries with laminar blood flow. In the areas of branching and bending of the arteries near the walls, vortex flows were formed, leading to mechanical stress on the walls, which was accompanied by pathological effects. These flows were characterised by low and low/oscillatory WSS (Cecchi et al., 2011; Morbiducci et al., 2016). A study conducted on isolated segments of blood vessels through which LDLs flowed demonstrated that the transport of LDLs into the vascular wall increased with a decrease in WSS and, on the contrary, decreased with an increase in WSS (Colic et al., 2015). Patient-specific modelling of the subendothelial accumulation of LDLs in the stenotic right coronary artery also showed an inverse relationship between the distribution of WSS and the accumulation of LDLs (Sakellarios et al., 2013). The zone of recirculating flow and low WSS corresponded to the maximum accumulation of LDLs, and in areas of high WSS, the accumulation of LDLs was low.

To localise the segments of arteries with low and low/oscillatory WSS and monitor the transformation of atherosclerotic plaques into a stable or unstable phenotype, computer modelling of blood flow in the vessels is being developed. This will facilitate the identification of patient-specific fields and gradients of blood flow rates depending on the geometry of the vessels (Soulis et al., 2006; Timmins et al., 2015, 2017; Hung et al., 2016). To solve these issues, the Navier–Stokes equations for an incompressible viscous fluid are used. To reconstruct the geometric shape of the vessels, intravascular ultrasound methods are used as well as X-ray microcomputer tomography with the use of contrast agents (Nebuloni et al., 2013; Xing et al., 2016). The ANSYS Fluent, OpenFOAM, FLUENT 6.0 and other software packages are widely used to conduct calculations via mathematical models of stationary and unsteady blood flows in various areas of the arteries. Computer modelling of the distribution of WSS, which accounts for patient-specific data on the geometry of blood vessels, is of high value for clinical practice.

The molecules of cell adhesion and their interactions in the early stage of atherogenesis

OxLDLs in the subendothelial space as well as low and low/ oscillatory WSS cause pro-inflammatory activation of endothelial cells (ECs), which leads to the rolling of leukocytes in the circulating blood flow to the endothelium, their adhesion and transendothelial migration (TEM). The mediators of these critical events of the early stage of atherogenesis are cell adhesion molecules. These molecules are expressed on the surface of ECs and circulating blood cells (monocytes or leukocytes and platelets) and include platelet endothelial cell adhesion molecule-1 (PECAM-1); intercellular adhesion molecule-1 and -2 (ICAM-1, ICAM-2); vascular cell adhesion molecule-1 (VCAM-1); E-, L- and P-selectins; vascular endothelial (VE) cadherin; β1 and β2 integrins; proline-rich glycoprotein CD99 and junctional adhesion molecule-A (JAM-A).

Selectins (transmembrane glycoproteins) are expressed on the surface of ECs (E-selectin, ELAM-1, P-selectin), leukocytes (L-selectin) and platelets (P-selectin) (Carlos, Harlan, 1994). Early experiments conducted in a flow chamber with a laminar flow of monocytes on an EC monolayer (physiologically low WSS, the use of functionally blocking monoclonal antibodies to L-, P-, E-selectin, ICAM-1, VCAM-1, β1 and β2 integrins) showed that the rolling of the monocytes to the ECs, weak, reversible contact of the monocytes with ECs (initial adhesion) and slowing down of the rate along the endothelium determine the interactions of the L-selectin of the monocytes with glycoprotein ligands of the ECs and, to a lesser extent, the P-selectin of the ECs with the glycoprotein ligand PSGL-1 of the monocytes. E-selectin is not involved in the process (Luscinskas et al., 1994, 1996). Firm, irreversible adhesion of the leukocytes to the ECs occurs during the interaction of the leukocyte α4β1 (VLA-4) integrin with the endothelial immunoglobulin VCAM-1 (Luscinskas et al., 1994; Huo, Ley, 2001) and the interaction of the leukocyte αLβ2 (LFA-1, CD11a/CD18; Mac-1, CD11b/CD18) integrins with the endothelial immunoglobulin ICAM-1 (Luscinskas et al., 1994; Sigal et al., 2000; Huo, Ley, 2001).

OxLDLs and lysophosphatidylcholine (a component of oxLDLs) induce the expression of ICAM-1 and VCAM-1 on the surface of cultured ECs and stimulate monocyte adhesion (Kume et al., 1992; Amberger et al., 1997). A physiologically low WSS, created by the flow of leukocytes (monocytes, neutrophils, lymphoblasts, lymphocytes) on the EC monolayer, generates upward docking structures on the endothelium that contain ICAM-1 and VCAM-1 clusters within 1–2 minutes. These clusters surround the leukocytes and function as an anchor for them (Barreiro et al., 2002; Carman et al., 2003). In turn, the structures of VCAM-1 and ICAM-1 that surround the leukocytes stimulate the formation of lateral linear tracks of leukocyte β1 (VLA-4) and β2 (LFA-1) integrins that are oriented parallel to the ICAM-1 and VCAM-1 clusters (Carman, Springer, 2004). Moreover, 90% of the leukocytes that are surrounded by the VCAM-1 and ICAM-1 clusters transmigrate to the subendothelial space, and the suppression of VCAM-1 and ICAM-1 via inhibitors (BARTA-AM, colchicine, toxin-B) significantly suppresses TEM. Regardless of the TEM pathway (the paracellular pathway between the ECs, the transcellular pathway through the ECs), the TEM process is associated with the formation of a cupped traction structure by the VCAM-1/VLA-4 and ICAM-1/LFA-1 interactions of endothelial and leukocyte cells, which guide and facilitate TEM (Carman et al., 2003).

VE-cadherin plays an important role in the TEM of leukocytes. VE-cadherin is only expressed in ECs, localised mainly in the intercellular contacts and plays an important role in the intercellular adhesion and barrier functions of the ECs (Garrett et al., 2017). The adhesion of leukocytes to the endothelium in the lateral intercellular contacts, preceding TEM, induces the formation of gaps in the intercellular distribution of VE-cadherin and the components of the VE-cadherin complex (α-, β-, ϒ-catenin, p120-catenin) at the sites of adhesion or transmigration of the leukocytes. The gaps are formed as a result of lateral displacement of VE-cadherin in the membrane and facilitate the TEM of the leukocytes. Following the completion of TEM, VE-cadherin moves in the opposite direction and closes the gaps (curtain opening and closing effect) (Allport et al., 2000; Shaw et al., 2001). The lateral displacement of VE-cadherin in the membrane most likely occurs due to the destabilisation of the VE-cadherin bond with the actin cytoskeleton by the following mechanism: the ICAM-1 and VCAM-1 clusters in the sites of intercellular adhesion of the leukocytes to the ECs induce the intracellular activation of the Src and Pyk2 tyrosine kinases and the phosphorylation of Tyr658 and Tyr731 of the cytoplasmic domain of VE-cadherin, which are involved in the low-affinity binding of VE-cadherin to p120- and β-catenin, respectively. The weakening of these interactions disrupts the VE-cadherin bond with the actin cytoskeleton, destabilises the VE-cadherin or VE-cadherin cell-cell interactions and facilitates the lateral movement of the phosphorylated VE-cadherin in the membrane (Allingham et al., 2007). p120-catenin regulates the phosphorylation of VE-cadherin and the paracellular TEM of leukocytes via a competition mechanism with the activated Src and Pyk2 tyrosine kinases: the overexpression of p120-catenin in the ECs leads to the absence of gaps in the distribution of VE-cadherin and the blocking of the TEM of the leukocytes (Alcaide et al., 2008).

The glycoprotein PECAM-1 (CD31) plays an important role in the TEM of leukocytes. In ECs, PECAM-1 is mainly localised in the intercellular contacts. The homophilic interactions of this protein with adjacent ECs occur through the extracellular Ig-like domains IgD1 and IgD2 (Paddock et al., 2016). PECAM-1 is also present in a distinct membranevesicular recycling compartment adjacent to the lateral border membrane of the ECs (Mamdouh et al., 2009). In the resting endothelium (in the absence of adhesive leukocytes), there is constitutive membrane traffic between the lateral cell border and the membrane-vesicular compartment (known as the lateral border recycling compartment, LBRC) (Mamdouh et al., 2003, 2008). In the presence of adhesive leukocytes on the endothelium, directed kinesin-dependent migration of the PECAM-1-bearing LBRC membrane along the microtubules of the ECs to the sites of para- and transmigration of the leukocytes occurs, in addition to the surrounding of the leukocytes by the LBRC membrane. The LBRC provides non-ligated PECAM-1 and CD99 in the ECs to interact with the homophilic ligands (PECAM-1 and CD99, respectively) in the leukocytes and initiates the signals for further recruitment of the LBRC as the leukocytes move through the endothelial layer. The antibodies to PECAM-1 and CD99 block the TEM of the leukocytes (Mamdouh et al., 2008, 2009). The recruitment of the LBRC to paracellular TEM sites precedes the formation of gaps in the intercellular distribution of VE-cadherin and is necessary for the formation of these gaps (Gonzalez et al., 2016). In ApoE–/– PECAM-1–/– mice, the load of plaques in the areas of the carotid artery with a low and low/oscillatory WSS was significantly less than in the control ApoE–/– mice (Harrison et al., 2013). A study on the relationship of single nucleotide polymorphisms (Val125Leu, exon 3; Asn563Ser, exon 8; Arg670Gly, exon 12) in functionally important domains of PECAM-1 in patients who were at risk of developing coronary heart disease and myocardial infarction showed that Arg670Gly substitution can be a homozygous protector for the development of myocardial infarction. This substitution is localised close to Tyr663, the phosphorylation of which, under low WSS conditions, initiates the signaling pathway of activation of the key transcription factor NF-κB for the expression of cell adhesion molecules (Sahebkar et al., 2013). Val125Leu and Asn563Ser substitutions are not associated with the risk of coronary heart disease (Xia et al., 2015).

Integrins also play an important role in cell adhesion. The integrins are a large family of receptors that are localised in the plasma membrane and consist of 18 α and 8 β subunits that form 24 different heterodimers. The extracellular domains of the integrins interact with ECM proteins (collagens, CL; fibronectin, FN; laminins, LN; vitronectin, VN; etc.) and ligands (for example, VCAM-1) on the surface of other types of cells, causing cell-substratum or cell-cell adhesion. The integrin-ligand interactions induce the activation of a variety of signaling pathways that modulate cellular behaviour, including proliferation, shape, motility, survival or apoptosis, differentiation, protein phosphorylation, cytoskeleton organisation and gene expression. Many integrins are expressed in an inactive state on the cell surface since the membrane-proximal highly conserved sequences of the cytoplasmic domains of the α and β subunits form a structural constraint that locks the conformation of integrins in an inactive, low-affinity state. The activation of integrins is often induced by intracellular signals and regulatory factors that act on the cytoplasmic domains, as well as phosphorylation. This alters the affinity of integrins for ligands through conformational changes in their extracellular domains, as well as clustering (Hynes, 2002).

The adhesion of leukocytes to the endothelium and their infiltration into the subendothelial space is enhanced by cytokines, chemokines and other factors. Thus, monocytic chemotactic protein-1 (MCP-1) (produced by macrophages and vascular wall cells) and interleukin-8 (IL-8) (produced by macrophages) are involved in the delay of peripheral circulation monocytes and their adhesion and migration into the arterial intima via interaction with monocyte receptors belonging to the CCR2 and CXCR2 types, respectively (Peters, Charo, 2001; Charo, Taubman, 2004). IL-9, secreted mainly by CD45+CD3–CD19– leukocytes in АпоЕ–/– mice, stimulates the expression of VCAM-1 in the ECs of the aorta of mice through interaction with the IL-9 receptor (IL-9R) and activation (phosphorylation) of the signaling protein and transcription activator STAT3 (Zhang et al., 2015). In the atherosclerotic aortas of АпоЕ–/– mice, a high level of IL-17A expression was observed, as well as numerous IL-17A-producing CD4+ T helper 17 (Th17) and γδ+ T cells. IL-17A initiates the production of several cytokines and chemokines by aortic cells, in particular, the pro-inflammatory chemokine CXCL1, which activates peripheral circulation monocytes and stimulates their adhesion and migration to the aortic wall (Erbel et al., 2014). Pro-atherogenic IL-17C, expressed mainly by aortic SMCs in АпоЕ–/– mice, is involved in the recruitment of T cells and macrophages into the aortic wall (Butcher et al., 2016). TGF-β-(H2O2-) inducible clone 5 (His-5), expressed on the surface of ECs and SMCs, is involved in the formation of structures that are similar to the microvilli on the surface of ECs, which enhance the adhesion of monocytes to the ECs (Arita-Okubo et al., 2015).

Transcription factor NF-κB is a key regulator of the gene expression of cell adhesion molecules under conditions of physiologically low and low/oscillatory wall shear stress

The transcription factor NF-κB positively regulates the expression of cell adhesion molecules with the participation of other transcription factors and coactivators. The VCAM-1 gene promoter has two NF-κB sites that are required for transcription activation (Neish et al., 1992). The C/EBP and NF-κB sites were identified in the ICAM-1 gene promoter, and mutations in the latter completely suppress the activation of the ICAM-1 promoter (Ledebur et al., 1995). The ELAM-1 gene promoter includes a CRE/ATF site, three NF-κB sites and three HMGI(Y) sites; two of the HMG I(Y) sites are located within the NF-κB sites (the interaction of HMG I(Y) and NF-κB with small and large DNA grooves, respectively). All three NF-κB sites are required for promoter activation and enhance the affinity of NF-κB and ATF-2 for the promoter (Whitley et al., 1994). The promoter of the MCP-1 gene (monocyte chemoattractant protein-1) contains the NF-κB site and the AP-1 site, which are necessary for maximum induction of the promoter (Martin et al., 1997). The core element GAGACC (SSRE) was identified in the pro-atherogenic platelet growth factor (PDGF) promoter that stimulates the proliferation and migration of SMCs (Resnick et al., 1993); it was found to interact with NF-κB (Davis et al., 2003).

In ECs, the most common p50/p65 (RelA) heterodimer is NF-κB. It is well known that, in the cytoplasm, latent NF-κB is associated with an inhibitor of IκB (mainly IκBα) and is inactive. When ECs are stimulated by cytokines TNFα, IL-1 or bacterial lipopolysaccharide (LPS) that interact with specific EC receptors, signaling pathways are activated, leading to the activation of the IκB kinase (IKK complex), which specifically phosphorylates IκB. After the ubiquitination of IκB and its proteasomal degradation, free NF-κB is translocated into cell nuclei (factor activation), interacts with elements of the DNA major groove and activates the expression of genes involved in the immune response, cell survival, carcinogenesis and inflammation (with the participation of other transcription factors and coactivators) (Oeckinghaus et al., 2011; Yu et al., 2015). Early studies demonstrated that active NF-κB and ICAM-1 were present in ECs, macrophages and SMCs in atherosclerotic plaques of arteries of deceased patients but were absent in the intima or media of the healthy arteries of those patients (Brand et al., 1996). OxLDL triggers in the ECs in vitro and in vivo signaling pathways for IKK complex activation; this includes the activation of focal adhesion kinase (FAK) and ribosomal S6 kinase (RSK) and leads to NF-κB activation, VCAM-1 expression and monocyte adhesion to ECs (Yurdagul et al., 2016). Using the model flow channel system (Frangos et al., 1985) that is created by a laminar pulsating flow of the culture medium containing monocytes, in which an average physiologically low and uniformly distributed WSS acts on the EC monolayer, it was shown that a mechanical stimulus (physiological low WSS) activates the IKK complex and NF-κB and stimulates VCAM-1 expression and monocyte adhesion to the ECs (Mohan et al., 1999). A model spatial gradient of averaged low/oscillatory WSS, close to the gradient of WSS in arterial branchings and bends, resulted in a more efficient activation of NF-κB compared to uniformly distributed low WSS (Nagel et al., 1999).

High activation of NF-κB was observed in ECs that were cultivated based on a model of the calculated WSS profile in a site of the human carotid sinus (atherogenic region, averaged low/oscillatory WSS) compared with a model of the calculated WSS profile in the distal segment of the carotid artery bifurcation (atheroprotective region, high average WSS) or cells at rest (Dai et al., 2004). A study of the transcriptional expression of the VCAM-1 endothelium from the nature of haemodynamics and calculated profiles of WSS in the segments of the left and right coronary arteries showed that low/ oscillatory WSS at the outer wall of the bifurcation of the left anterior descending artery (atherogenic region) induces a significantly higher expression of VCAM-1 compared to a straight region of the right artery (laminar flow, high WSS), in which atherosclerotic plaques are largely unformed (O’Keeffe et al., 2009). The mapping of the endothelial expression of NF-κB/IκB and the activation of NF-κB in areas of the aortic arch of mice with high (high probability, HP; internal arch of the aortic arch) and low (low probability, LP; external arch of the aortic arch) probability of atherosclerotic plaque formation demonstrated that in the HP region with low/oscillatory WSS (Suo et al., 2007), the expression of NF-κB/IκB and the activation of NF-κB significantly exceeded those in the LP region (Hajra et al., 2000). S. Cuhlmann et al. (2011) also detected increased expression of the RelA subunit NF-κB and a higher nuclear localisation compared with the LP region in the HP region of the aortic arch of mice. Moreover, this was correlated with increased expression of VCAM-1 and the accumulation of CD68+ macrophages in the HP region

The gap-junction protein connexin 40 (Cx40), expressed in ECs under low WSS conditions, whose cytoplasmic C-terminus interacts with IκBα and inhibits its phosphorylation, is a negative regulator of NF-κB activation in areas with low WSS (Denis et al., 2017). Conversely, mechanosensitive phosphatidic acid phosphatase (PPAP2B) regulates NF-κB activity, expression of adhesion proteins, monocyte adhesion and TEM under high WSS. PPAP2B shows increased expression in ECs under atheroprotective flow characteristics (high WSS, laminar flow) and shows decreased expression under atherogenic characteristics (low WSS, disturbed flow). PPAP2B hydrolyses lysophosphatidic acid (LPA), a pro-atherogenic and thrombogenic glycerophospholipid in the blood and, thereby, blocks signaling pathways activated by the interaction of LPA with cellular LPA receptors (LPAR). LPA-LPAR1 interaction activates the Rho kinase-NF-κB signaling pathway and the subsequent transcriptional expression of ICAM-1, VCAM-1 and E-selectin in ECs (Shimada et al., 2010; Wu et al., 2015).

LPA-LPAR1/2 interaction leads to an increase in the contractility of ECs and the permeability of the endothelial monolayer (Wu et al., 2015). LPA is an extracellular signaling molecule that is capable of interacting with at least six G protein LPA-LPAR1/2 interaction leads to an increase in the contractility of ECs and the permeability of the endothelial monolayer (Wu et al., 2015). LPA is an extracellular signaling molecule that is capable of interacting with at least six G protein- coupled cellular receptors, initiating intracellular signaling cascades (Yung et al., 2014) and exerting multiple effects on blood cells (platelets, monocytes) and vascular wall cells (ECs, SMCs). Therefore, in platelets, LPA induces a change in the shape, aggregation, and formation of platelet-monocyte aggregates. In ECs, LPA induces cell migration, expression of VCAM-1, ICAM-1, E-selectin, chemokines (CXCL1), formation of actin stress fibres and cell contraction. In SMCs, LPA induces cell contraction, migration and proliferation. Additionally, LPA accumulates in the lipid-rich core of atherosclerotic plaques, and, when ruptured, enters the bloodstream and activates platelets, leading to the formation of blood clots (Schober, Siess, 2012). Endothelial NO synthase (eNOS) is also expressed under physiologically high WSS. Under these conditions, the eNOS promoter is activated through the interaction of NF-κB with the GAGACC element (Davis et al., 2003). eNOS produces NO, an inducer of IκBα expression. NO stabilises the NF-κB∙IκBα heterotrimer in the cytoplasm and induces IκBα translocation into cell nuclei, leading to the inactivation of NF-κB and the termination of NF-κB-mediated transcription (Spiecker et al., 1997). Moreover, NO inhibits IκB kinase (IKK complex), a positive regulator of NF-κB activation (Yurdagul et al., 2013).

Mechanosensitive signaling pathways that control the transcriptional expression of the RelA subunit of NF-κB in endothelial cells

The WSS, created by the blood flow to the walls of the arteries, activates mechanosensitive signaling pathways in the ECs. Amechanosensor localised in the cell membrane perceives a mechanical stimulus (WSS) and triggers intracellular signaling that activates specific transcription factors, which regulate the transcriptional expression of proteins. The internal bend of the aortic arch and the areas of arterial bifurcation, where low and low/oscillatory WSS are realized, are associated with the localisation of atheroma. In the same areas, increased expression of active c-Jun N-terminal kinase 1 (JNK1), which belongs to mitogen-activated protein kinases (MAPK), was revealed (Zakkar et al., 2008), as well as increased expres-sion and nuclear localisation of the RelA subunit of NF-κB (Cuhlmann et al., 2011). The hypothesis that physiologically low WSS regulates the expression of RelA and NF-κB target genes (VCAM-1 and others) through the JNK1-dependent pathway was proved by modelling the haemodynamics of the carotid artery in mice. The implantation of a tapered cuff around the mouse carotid artery generates a laminar blood flow with a low velocity and low WSS upstream of the cuff and disturbed, low velocity and low/oscillatory WSS downstream of the cuff (Cheng et al., 2006; Xing et al., 2016). This approach demonstrated that increased expression of RelA and activated JNK1 is realised in regions with low and low/oscillatory WSS. The transcription factor ATF2 is activated by JNK1 (phosphorylation) and interacts with the RelA promoter sites to activate the promoter. Thus, in areas with low/oscillatory WSS, the JNK-ATF2-RelA signaling pathway is implemented, which stimulates the expression of RelA and NF-κB target genes (VCAM-1). The pathway also stimulates the accumulation of CD68+ macrophages, which are an indicator of the development of arterial inflammation (Cuhlmann et al., 2011).

The signaling pathway for the transcriptional expression of RelA upstream of JNK1 includes the activation of integrins, which is initiated by the stimulation of the cell surface mechanosensory complex and an integrin-dependent signaling cascade, leading to the activation of JNK1. The cell surface mechanosensory complex includes the PECAM-1 and VE-cadherin receptors and the vascular endothelial growth factor 2 receptor (VEGFR2), which belongs to the receptor tyrosine kinase subfamily (Tzima et al., 2005). In this complex, PECAM-1 is a key mechanosensitive signaling molecule that perceives a mechanical signal and converts it into a chain of intracellular biochemical reactions. WSS, acting on the extracellular domain of PECAM-1, affects the conformation of the cytoplasmic domain of PECAM-1 and the availability of Tyr663 and Tyr686 of this domain for phosphorylation. Phosphorylation is conducted by membrane-bound Fyn tyrosine kinase (family of Src tyrosine kinases), which is localised in intercellular contacts near PECAM-1 (Chiu et al., 2008). PECAM-1/PECAM-1 intercellular EC interactions through the extracellular domains of PECAM-1 (Paddock et al., 2016) are required for efficient phosphorylation and the triggering of intracellular mechanosensitive signaling (Chiu et al., 2008; Snyder et al., 2017). Activated PECAM-1 and VE-cadherin (which functions as an adapter and is associated with VEGFR2) facilitate the phosphorylation of VEGFR2 at Tyr801 and Tyr1175 by the Src tyrosine kinase. In turn, the phosphorylated VEGFR2, through direct interaction with the regulatory p85 subunit of the phosphatidylinositol-3-OH kinase (PI(3)K), phosphorylates PI(3)K (Tzima et al., 2005).

The activated PI(3)K stimulates the conformational activation of integrins through the conservative pathway of the association of phosphatidylinositol-3,4,5 triphosphate (PI(3)K product) with pleckstrin homology (PH) domains of cytohesin- 1 or cytohesin-like proteins. This is followed by the translocation of these proteins to the plasma membrane and their association with the cytoplasmic domains of the β subunits of integrins, which is realized in various types of cells. This interaction leads to conformational changes in the extracellular domains of the α and β subunits of integrins and an increase in their affinity for specific ECM proteins, as well as clustering (Hughes, Pfaff, 1998; Hynes, 2002). In athero-resistant areas of arteries, the ECM is rich in CL (IV) and LN. However, in atherogenic regions (low/oscillatory WSS conditions), the ECM is enriched in the pro-inflammatory proteins FN and FG (Orr et al., 2005; Feaver et al., 2010; Collins et al., 2014). Antibodies specific to ligated β1 and β3 integrins were used to show that, under conditions of low/oscillatory WSS, there is an increase in the binding of α5β1 integrin to FN (ligand of α5β1, αvβ3, αvβ5 integrins) and of αvβ3 integrin to VN. Thus, the conformational activation of integrins and the dynamic formation of new bonds of integrins with specific ligands occur – ECM proteins are realized (Jalali et al., 2001; Tzima et al., 2001).

The composition of the ECM and the ligation of integrins by the ECM proteins activate many intracellular signaling cascades – in this case, the Shc-Grb∙Sos-Ras-MAPK signaling pathway. The cellular adapter protein Shc, activated under conditions of low/oscillatory WSS by tyrosine kinases Src and VEGFR2 close to the EC contacts (phosphorylation of Tyr239/240), forms an early unstable complex Shc∙VEGFR2∙VE-cadherin (Liu et al., 2008). Next, a stable complex Shc with an αvβ3 integrin that is ligated with FN or VN is formed (Chen et al., 1999; Jalali et al., 2001; Liu et al., 2008). Additionally, Shc associates with the β1 and β5 integrins that are ligated with FN and VN (Chen et al., 1999; Jalali et al., 2001). In this manner, Shc coordinates the intercellular contact proteins (VE-cadherin) and integrin-ECM interactions under low/oscillatory WSS conditions. Membrane-associated small G proteins Ras (small GTPases Ras) function cyclically between active Ras∙GTP and inactive Ras∙GDP forms, which are a molecular switch of an intracellular signal in response to an extracellular stimulus (Johnson, Chen, 2012). The complexes of phosphorylated Shc with the αvβ3, β1 and β5 integrins formed on the cytoplasmic side of the plasma membrane are accompanied by the association of Shc with the cytoplasmic Grb2∙Sos complex of the growth factor receptor-bound protein 2 (Grb2) and the guanine nucleotide exchange factor Sos. This complex stimulates the rate of exchange of the GDP associated with Ras, on the GTP (the interaction of Shc with Grb2∙Sos recruits Sos to the cytoplasmic side of the plasma membrane, providing the activation of membrane-localised Ras∙GDP) (McCormick et al., 1993). In turn, activated Ras stimulates cytoplasmic Raf kinase (kinase kinase MAPK, MAPKKK, MEKK) and recruits Raf to the inner surface of the plasma membrane through direct interaction with its regulatory domains and the subsequent phosphorylation of four sites of the kinase domain (Dumaz, Marais, 2005). Raf, activated via a MAP kinase cascade, activates MAPK (JNK) (Davis, 2000).

However, the activation of Shc and membrane-associated heterotrimeric G proteins (that consist of α, β, and ϒ subunits) leads to the stimulation of Ras under the conditions of the action of WSS on the cells. In the absence of WSS, G proteins are activated via association with ligand-activated receptors: the α subunit exchanges the GDP bound to it for GTP and dissociates from the βϒ dimer. The α∙GTP and βϒ complexes become mediators of cellular event signaling until the α subunit restores its inactive GDP-bound state (since it has GTPase activity.) The reassociation of α∙GDP with the βϒ dimer provides an inactive Gαβϒ heterotrimer that is capable of entering a new activation cycle (Simon et al., 1991). Alterations in the physical properties of membranes under WSS conditions (including the ordered configuration of the phospholipid bilayer [lipid order], fluidity and cholesterol content) affect the conformation and functions of membrane-associated proteins and, as a result, the signaling pathways activated by these proteins (Yamamoto, Ando, 2018).

Purified heterotrimeric G proteins in phospholipid liposomes loaded with [ϒ-32P]GTP were shown to be activated by the action of physiological levels of WSS on liposomes (Gudi et al., 1998). Within 1 second, a physiologically low WSS activated the Gαq/11 and Gαi3/0 subunits of G proteins in ECs (Gudi et al., 1996). The activated Gαq and Gβϒ subunits dissociated from them under conditions of a WSS gradient in a physiologically low range and initiated Ras activation and downstream MAPK signaling (Gudi et al., 2003). Moreover, two primary basic mechanosensors, Gαq/11 and PECAM-1, establish a mechanosensitive Gαq/11∙PECAM-1 complex, which is formed under conditions of laminar flow (physiological high WSS) in vivo (atheroprotective straight region of the descending aorta of mice) and in vitro. Under oscillatory flow conditions (low/oscillatory WSS), leading to the activation of G proteins, this complex is rapidly (within 30 seconds) destroyed (Otte et al., 2009). The formation of the Gαq/11∙PECAM-1 complex involves PECAM-1 extracellular Ig-like domains 2 and 3, as well as a Gαq/11 interacting receptor that associates with Gαq/11 and PECAM-1 Ig-like domains 2 and 3 and serves as a bridge in the formation of the complex (Yeh et al., 2008). The PECAM-1∙Gαq/11 complex also includes heparan sulfate proteoglycan, which associates with the Ig-like domain 3 of PECAM-1 and mediates the formation of the PECAM-1∙Gαq/11 complex (Paz et al., 2014).

## Conclusion

Computer modelling of the blood flow in the arteries makes it possible to determine the most atherogenic areas of the arteries, which are characterised by low and low/oscillatory WSS. The transcription factor NF-κB and the cell adhesion molecules ICAM-1, VCAM-1 and E-selectin are the earliest markers of atherogenesis. Therefore, the processes involved in the expression of these proteins under the conditions induced by a mechanical stimulus (low and low/oscillatory WSS) on endothelial cells are of great interest. This review presents an analysis of numerous studies that demonstrated how the activation of membrane-bound proteins that perceive a mechanical stimulus (low and low/oscillatory WSS) triggers a cascade of biochemical reactions that lead to the transcriptional expression of NF-κB, a key regulator of the expression of cell adhesion molecules. This review also describes in detail the mechanisms of interaction between the endothelial cell adhesion molecules and blood leukocytes that are responsible for adhesion and the subsequent TEM of leukocytes during the initial stage of atherogenesis. Studying the molecular processes involved in the initiation and development of atherosclerosis is extremely important for the development of an effective defence against this disease.

## Conflict of interest

The authors declare no conflict of interest.
